# Editorial: Biomarkers and Clinical Indicators in Motor Neuron Disease

**DOI:** 10.3389/fneur.2019.01318

**Published:** 2019-12-12

**Authors:** Peter Bede, Pierre-Francois Pradat

**Affiliations:** ^1^Computational Neuroimaging Group, Trinity College Dublin, Dublin, Ireland; ^2^Department of Neurology, Pitié-Salpêtrière University Hospital, Paris, France; ^3^Sorbonne University, CNRS, INSERM, Biomedical Imaging Laboratory, Paris, France

**Keywords:** ALS, MND, PLS, SMA, SBMA, PPS, PMA, biomarker

Motor neuron diseases (MNDs) encompass a range of progressive neurodegenerative conditions with heterogeneous clinical presentations, disability profiles, prognosis, and age of onset. The umbrella term MND typically includes amyotrophic lateral sclerosis (ALS) (1), primary lateral sclerosis (PLS) (2, 3), progressive muscular atrophy (PMA), hereditary spastic paraplegia (HSP), spinal muscular atrophy (SMA) (4, 5), spinal and bulbar muscular atrophy (SBMA) (6), and rare conditions such as monomelic amyotrophy (MMA), juvenile muscular atrophy of distal upper extremity (JMADUE) (7), Mill's disease (8), ALS-FTD complex (9), and progressive bulbar palsy (PBP) (10). Despite the diversity of the clinical phenotypes, MNDs share a number of fundamental traits such as a long presymptomatic phase (11), insidious onset (12), considerable diagnostic challenges (13, 14), relatively low incidence (15, 16), extra-motor (Christidi et al.; Christidi et al.) and extra-neurological manifestations (6), relentless progression (17, 18), multidisciplinary care needs (19, 20), and lack of effective disease modifying therapies. These core similarities justify the discussion of various MNDs in a dedicated collection of articles and offer the opportunity to exchange research ideas between centres focusing on specific MNDs. There are other shared challenges across the MND spectrum, which are particularly relevant for therapy development, chief of which is the lack of validated biomarkers to serve as outcome measures in clinical trials. Pharmaceutical trials in MNDs mostly rely on functional rating scales and survival instead of objective, observer-independent markers which reflect on the underlying pathology of the condition (1, 21, 22).

## Introduction

The dual relevance of biomarker development in MND lies in the characterization of dynamic pathological processes and its application to individualized patient care. From an academic perspective, biomarkers have the potential to elucidate the role of specific pathophysiological mechanisms, such as inflammation, cortical hyperexcitability, inhibitory dysfunction, cell to cell propagation and anatomical patterns of vulnerability. From a clinical standpoint, validated biomarkers have the potential to confirm an earlier diagnosis, thus enabling recruitment into clinical trials at an earlier stage. The key advantage of biomarkers however is their potential monitoring role in clinical trials; tracking disease progression *in vivo* and potentially detecting response to therapy. Biomarkers may also act as prognostic indicators which are indispensable both for patient stratification in clinical trials as well as individualized patient care.

The academic and clinical importance of biomarker development in MND is universally recognized by various scientific consortia and is regarded as a strategic funding priority by MND charities and funding agencies around the world. MNDA, ALSA, ARSLA, NISALS, NEALS, CALSNIC, RMN, JPND, IMNDA, SPF etc. are just some of the many organizations actively engaging in the development of multicenter data repositories, and establishing biobanking infrastructures for MND. Due to the limitations of single markers, it is generally accepted that a panel of biomarkers will most likely aid clinical management, guide care planning, and serve as monitoring markers in clinical trials. It is also increasingly clear that precision individualized therapies will be needed for specific phenotypes and genotypes instead of a “one-drug-for-all” approach.

The main themes of MND biomarker research include “wet biomarkers” which focus on disease-specific biofluid profiles and “dry biomarkers” such as electrophysiological and neuroimaging measures. Hypothesis-driven, targeted and high-throughput methods are both widely used in the so-called “omics” approaches: metabolomics, proteomics, lipidomics, and transcriptomics. One of the alluring aspects of international collaborations is that MND centers around the world have unique local expertise profiles which complement synergistically the skillset of other centers. Therefore, single ALS centers are in a position to provide authoritative reviews on specific aspects of biomarker research efforts. The editors of this collection are grateful for the expert contribution of 37 renowned research centers from around the globe. The 37 research papers included in this Research Topic discuss specific aspects of biomarker development in motor neuron diseases and embrace the diversity of MND phenotypes from SBMA to ALS-FTD. While the methodological focus of the papers differs depending the expertise profile of the authors, there is a cohesive theme among the papers to appraise biological, molecular, electrophysiological, and radiological markers which may potentially serve as pragmatic clinical indicators confirming the diagnosis, predicting the prognosis, detecting response to therapy or track longitudinal neurodegenerative changes. Beyond the practical relevance of ascertaining and quantifying pathological changes *in vivo*, biomarkers in MND also provide considerable academic insights such as the exploration of presymptomatic changes (23, 24), the description of genotype-associated signatures (25, 26), the delineation of natural disease trajectories (11, 27), the characterization of low-incidence phenotypes (2, 28, 29), confirmation of epigenetic and epidemiological factors (30, 31), and deciphering the pathological substrate of clinical symptoms [Finegan et al.; (32, 33)].

One of the commonest adult-onset motor neuron diseases is amyotrophic lateral sclerosis (ALS) which is an archetypical neurodegenerative condition with a presumed long presymptomatic phase (34), considerable delay between symptom onset and definite diagnosis (35), significant individual variations in disability profiles (Yunusova et al.), unrelenting motor decline (36), widespread non-motor symptoms (37, 38), and complex genetics (39, 40). In this collection of papers (https://www.frontiersin.org/research-topics/7659/biomarkers-and-clinical-indicators-in-motor-neuron-disease) wet and dry biomarkers are equally represented. “Wet” biomarkers typically refer to spinal fluid, serum or tissue-based indicators, whereas “dry” markers indicate non-invasive radiological, neuropsychological and or clinical indicators (41).

## Wet Biomarkers

Two papers are dedicated to the academic and biomarker role of micro RNAs; Joilin et al. discuss the diagnostic and prognostic utility of specific microRNAs and Rob Layfield's group propose the targeted study of four miRNAs; hsa-miR-124-3p, hsa-miR-127-3p, hsa-let-7a-5p, and hsa-miR-9-5p as particularly promising biomarkers (Foggin et al.). Tan and Guillemin discuss the potential biomarker role of kynurenine pathway metabolites in ALS, as these are involved in inflammation, excitotoxicity, oxidative stress, immune responses, and energy dysregulation. Chen et al. base their study on the inflammatory hypothesis of ALS etiology and not only demonstrate increased IL-6 levels in astrocyte-derived exosomes in ALS patients but identify associations with rate of progression. Dr. Duguez's group meticulously reviews the literature and suggest a multi-tissue biomarker panel encompassing markers of motor neuron integrity (pNFH and NF-L, cystatin C, Transthyretin), inflammation (MCP-1, miR451), muscle integrity (miR-338-3p, miR-206) and metabolism (homocysteine, glutamate, cholesterol). They argue that biomarker panels should reflect the multi-system, multi-tissue nature of ALS-pathophysiology (Vijayakumar et al.). As ALS is increasingly recognized as a metabolic disorder (42), De Aguilar provides an eloquent overview of metabolic markers with a particular focus on proposed lipid biomarkers. Kirk et al. elaborate on the metabolic spectrum of ALS from cellular to multi-organ systemic involvement. Dr. Blasco's team discusses advances in metabolomics and advocates for a pharmacometabolomic approach to evaluate individual response to therapy, to develop personalized treatments for ALS (Lanznaster et al.). Poesen and Van Damme review the diagnostic, monitoring and prognostic role of neurofilaments in ALS.

## Clinical Indicators and Therapeutic Strategies

Zhang et al. draw the reader's attention to comorbid extra-neurological manifestations in ALS, such as autoimmune syndromes. Lule et al. contribute an authoritative review of the key determinants of quality of life in ALS, and underline the lack of a direct link between physical disability and quality of life. Professors Lule and Ludolph also emphasize the key ethical principles of supportive care in ALS which are centered on patient autonomy, dignity, beneficence and caregiver support (20, 43–47). Li Hi Shing et al. highlight the complex symptomatology of post-polio syndrome and discuss the etiology of under-researched symptoms such as fatigue. Finegan et al. perform a comprehensive review of the pathophysiology of pseudobulbar affect (pathological crying and laughing) which is one of the most prevalent and distressing symptoms of PLS and ALS, yet it remains surprisingly understudied (32, 33, 48). Chipika et al. undertake a systematic analysis of the most promising markers to track pathological progression *in vivo*, which may detect response to therapy in future clinical trials of ALS. Their primary perspective is the assessment of the pragmatic utility [Grollemund et al.; (14, 35, 49–51)] of emerging markers in pharmaceutical trials (Chipika et al.). Christidi et al. reviews the evidence for memory deficits in MND with a painstaking analysis of the available clinical (52), radiological (1) and post mortem literature (53). Professor Mioshi's group eloquently reviews the impact of cognitive and behavioral deficits in ALS on patients and caregivers drawing attention to an important aspect of ALS care which is relatively understudied (54, 55). The novelty of their analysis lies in the identification of viable non-pharmaceutical strategies to improve patient and caregiver well-being (Caga et al.). Grollemund et al. perform an in-depth analysis of the ever expanding literature of machine-learning in MND, and discuss the advantages and drawbacks of specific mathematical models. Professor de Carvalho's group gives an authoritative overview of respiratory markers and diaphragmatic neurophysiology in ALS (de Carvalho et al.). Professor Yunosova's group appraises the most commonly utilized clinical tools for assessing and monitoring bulbar dysfunction in ALS and advocate for the development and validation of novel assessment protocols (Yunosova et al.). Professor Kabashi's group gives an elegant overview of neuromuscular junction involvement in ALS and examines the evidence from animal models to clinical observations (Campanari et al.). Dr. Floeter's group discuss genotype-specific biomarker panels and presymptomatic alterations. They review candidate imaging, electrophysiology, and biofluid markers in patients with *C9orf72* hexanucleotide expansions (Floeter et al.). Christidi et al. discuss the clinical (38, 56–58), imaging (31, 36, 59, 60), and pathological correlates of cognitive and behavioral dysfunction in ALS giving specific screening and assessment recommendations. They describe which domains are most likely to be affected and review the impact of neuropsychological deficits on patients and their caregivers (Christidi et al.). Querin et al. evaluate monitoring strategies in spinal and bulbar muscle atrophy (SBMA) and discuss the spectrum of motor, extra-motor, and extra-neurological manifestations in detail. They give specific recommendations to screen for endocrine, cardiac and respiratory involvement (Querin et al.).

From a therapeutic viewpoint, Gouel et al. discuss the role of neurotrophic growth factors (NTF) in neuroprotection and neurorestoration. Professor Bogdahn's group give a real-life example of using biomarkers in a therapeutic trial of Granulocyte-colony stimulating factor (G-CSF). They evaluate the biomarker potential of serum cytokines in ALS and perform a meticulous analysis of MDC, TNF-beta, IL-7, IL-16, and Tie-2 levels in relation to clinical outcomes (Johannesen et al.).

## Dry Biomarkers

Electrophysiology is one of the most widely utilized clinical and research tools in motor neuron diseases [Proudfoot et al.; (5, 61, 62)]. Professor Kiernan's group provides an expert review of electrophysiological markers of upper and lower motor neuron degeneration and discuss the clinical value of specific neurophysiological indices (Huynh et al.). Wang et al. present an elegant electrophysiology study, investigating the neurophysiological substrate of the split-hand phenomenon. Imaging is another promising dry biomarker of ALS-associated degenerative change. In recent years imaging in ALS confirmed extensive extra-motor pathology in cerebellar (63, 64), extra-pyramidal (65, 66), subcortical (26, 67), hippocampal [Christidi et al.; (68, 69)], hypothalamic (42), brainstem [Yunusova et al.; (70)], and frontotemporal involvement [Christidi et al.; (71)]. Imaging in ALS also helped to decipher the pathological underpinnings of specific symptoms, such as pseudobulbar affect [Finegan et al.; (32, 33, 48)], compensatory changes (72), executive dysfunction (73), extrapyramidal manifestations (65), metabolic dysfunction (42), memory deficits (59, 74). Imaging in ALS has also been instrumental to link disability profiles to pathological TDP-43 burden patterns (36, 53, 75–78) and track progressive pathological changes [Chipika et al.; (11, 79)]. In this collection of papers, Fortanier et al. demonstrate how structural imaging data may be used to characterize alterations in connectivity patterns. Rajagopalan and Pioro elegantly demonstrate how clinically well-defined ALS sub-populations have distinctive neuroimaging signatures. Instead of the most commonly used quantitative techniques, such as diffusion tensor imaging (80, 81) they demonstrate the utility of alternative imaging cues on T2-wighted, Flair and proton density imaging (Rajagopalan and Pioro). Kalra a pioneer of MR spectroscopy, gives an eloquent overview of the achievements, practical utility and future applications of metabolic imaging in ALS. Professor Filippi and Dr. Agosta's research group contributed an expert review of diffusion imaging in ALS, discussing methodological advances, the contribution of network analyses, and the potential of DTI to track progressive pathological changes (Basaia et al.). Their observations also highlight the paradigm shift from the analysis of focal diffusivity changes (80, 81) to the assessment of network integrity (41, 82). Muller and Kassubek review the utility of diffusion tensor imaging in ALS with respect to detecting pathological TDP-43 burden *in vivo*. They describe how *in vivo* measurements may relate to pathological stages and provide an expert overview of the most frequently utilized analysis methods (Muller and Kassubek). The majority of imaging studies in motor neuron disease focus on cerebral pathology (72, 83–85), despite the pathognomonic involvement of the spinal cord in ALS [El Mendili et al. (86)], SBMA (6, 10), SMA (4, 5), PLS (2), juvenile muscular atrophy of distal upper extremity (7, 28, 87) and PPS (29). In this Research Topic, Professor Pradat's group gives a methodological update on advances in spinal imaging and outline future research directions (El Mendili et al.). Chew and Atassi discuss how PET radioligands unveil specific pathophysiological mechanisms such as neuroinflammation, metabolic changes, neuronal dysfunction, and oxidative stress and how PET may be utilized both in natural history studies and pharmaceutical trials. Professor Turner's group reviews the advances in functional imaging discussing the contribution of functional MRI, MEG and EEG studies to ALS research (Proudfoot et al.). Dr. Grosskreutz's group discusses the benefits of data sharing and gives an expert overview of the methodological and logistical challenges of data harmonization, hosting large data repositories, generating consortium bylaws and data protection policies (Steinbach et al.). Barritt et al. summarize some of the most exciting new imaging methods in MND and discuss emerging methods such as Neurite Orientation Dispersion and Density Imaging (NODDI) (88), and quantitative Magnetization Transfer Imaging (qMTi) and data analysis approaches such as Event-Based Modeling (EBM). A shared aspiration of both “wet” and “dry” biomarker studies is the transition from describing group-level observations to the precision categorization and tracking of individual patients (53, 76, 83, 84, 89–91).

## Conclusions

The ensemble of these articles showcases the determination, drive and momentum in motor neuron disease research worldwide. We are particularly proud that renowned research groups from Australia, France, China, Greece, United Kingdom, Ireland, United States, Canada, Germany, Belgium and Italy shared their unique perspective, methodological expertise and their vision for future research directions. The diversity of research strategies and the unrelenting enthusiasm of the various research groups give cause for optimism for the development of precision biomarkers, and ultimately, a cure for MND.

## Author Contributions

Both authors contributed equally to the drafting of this editorial.

### Conflict of Interest

PB is the patron of the Irish motor neuron disease association (IMNDA), the head of the computational neuroimaging group (CNG) in Trinity College Dublin, member of the steering committee of the Neuroimaging Society of ALS (NiSALS) and member of the biomedical research advisory panel of the UK MND association (MNDA). These affiliations had no impact on the opinions expressed herein. P-FP denies any commercial or financial relationships that could be construed as conflict of interest.
